# Assessment of seat-belt related injuries using the FORTIS forensic system

**DOI:** 10.25122/jml-2021-0069

**Published:** 2021

**Authors:** Nikita Bobrov, Ján Mandelík, Aleš Vémola, Alena Obrátilová, Bohumil Šejnoha

**Affiliations:** 1. Department of Forensic Medicine, Faculty of Medicine, University Hospital of P. J. Šafárik Košice, Košice, Slovak Republic; 2. Department of Transport Safety, The University of Security Management in Kosice, Kosice, Slovak Republic; 3. Institute of Forensic, Brno University of Technology, Brno, Czech Republic

**Keywords:** medicine, traffic accident, passengers, injuries, parametrization of injuries, damage to health, judicial proceeding

## Abstract

This study presents (1) a case of an injury to an unbelted passenger and (2) the possibilities of proving the occurrence of injuries to traffic accident participants. We demonstrate the case of an injury to a passenger who failed to fasten her seat belt, and question whether her injuries would have been equally serious if she had fastened her seat belt. Theoretical bases and methods for the interdisciplinary procedure of medical examiners using the PC Fortis program and technical analysts of traffic accidents using the PC Crash program are presented. Furthermore, individual practical steps are documented, the result showing that the injuries to the passenger would have occurred, but, to a minimum extent, i.e., 6.9% of the original injuries, which would have not exceeded the legal limit for damage to health.

## Introduction

In answering serious legal questions from investigators and courts, contemporary medicine has new possibilities for systemic interdisciplinary cooperation between forensic physicians and technical experts through a new method of injury quantification and localization, namely the FORTIS system developed by the Department of Forensic Medicine at the Faculty of Medicine of P. J. Safarik University.

The system responds to how to use a great deal of new information on the nature and extent of physical parameters resulting from the movement and contacts of a vehicle and participants in an accident, provided by simulation programs. Consequently, this gives new possibilities for taking evidence for investigations and legal assessments, which have not been possible so far. For instance, the exact specification of the extent of injury caused by a traffic accident due to changed circumstances can also include finding the difference in the degree of injury in a belted passenger compared to an unbelted one. From this point of view, this article provides information on the latest methods for proving the injuries of participants in traffic accidents.

Technical experts in road transport perform technical analyses of accidents in which participants are injured to clarify the course of an accident and make the cause of occurrence clear.

As every traffic accident is a combination of individual and unique physical parameters and phenomena, it is indisputable that injuries represent an individual and unique reflection of the physical violence affecting the participants in a traffic accident. Until now, a technical expert in road transport has had extremely limited possibilities to use the information on the extent and manner of injuries in a traffic accident participant, when dealing with the course of a traffic accident, even after using advanced simulation programs. The reason is that such information cannot be explicitly interpreted from a technical point of view to be used for the accident analysis itself [1]. Moreover, the form of the information provided does not correspond to the need of their technical assessment since it is usually a verbal description of injuries issued by a doctor. An expert in road transport does not have sufficient medical background to interpret this information correctly and accurately, which sometimes may lead to incorrect conclusions. Nevertheless, when assessing the importance of the information regarding the course of an accident, it is undeniable that the type, extent, and location of injuries of each traffic accident represent essential information. Suppose this does not correspond to the results of a technical analysis or a collision simulation, subsequently the derived course of the accident could be considered incomplete if not incorrect in some cases, or impossible and technically unacceptable in extreme cases.

In practice, however, there are cases where, to assess the degree of fault, it is necessary to determine the biomechanical response, which represents the extent of injuries, that could have occurred if some circumstances of the accident had been different. Probably, the most common case when such an assessment is required is a case of an unbelted passenger (driver, front-seat passenger, rear-seat passenger) where it is necessary to prove the extent of injuries the person in question would have suffered if he/she had fastened the seat belt and had been wearing it during the accident.

This is also the case of the presented injuries to the unbelted passenger caused by a drunk driver, which was processed at the request of the court, and which represents practical possibilities of how to use FORTIS medical system by doctors together with the simulation program used by technical experts. Improved methods of proving should become part of the transport policy in the European Union, and these should have a positive effect on driver discipline on European roads [7].

## Material and Methods

The FORTIS forensic system, which allows to parametrize individual injuries, and to locate the places of action of contact forces resulting in the occurrence of detected injuries (using the PC Fortis program), was used to deal with the case. This way, the extent of violence and the affected location on the body of an injured person for each injury are defined. Defined injuries may be technically assessed regarding the contacts in the accident simulation, while the extent of the injuries may be detected using the outputs of the PC Crash simulation program [6]. Thus, this procedure connects methods of forensic engineering and forensic medicine into a single interdisciplinary procedure while retaining the theoretical and practical resources of both disciplines. Consequently, this offers a more complex technical analysis of an accident and the possibility to determine the extent of injuries occurring in the accident simulation.

## Results

### Findings from the site of the accident

**Figure 1.**
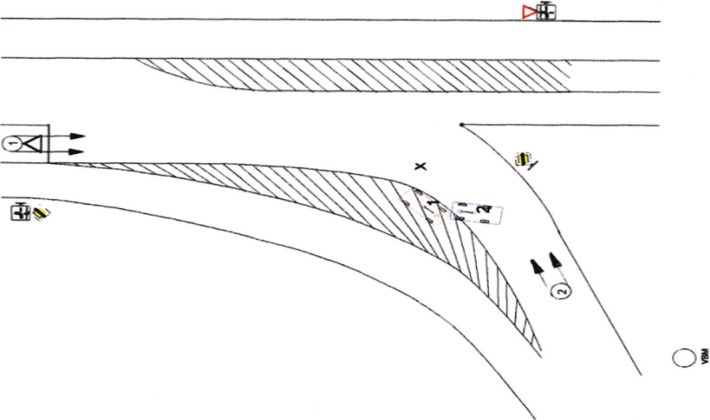
Plan of the traffic accident site.

The traffic accident occurred in the residential area, at a crossing, with poor visibility. Based on the inspection of the accident site, an investigator prepared the plan of the traffic accident site – Figure 1 with an indication of the approximate construction condition of the accident site, traffic situation, direction of movement of the vehicles – Sedan (marked as 1) and Pick-Up (marked as 2), collision site (X), and final positions of the vehicles after collision.

**Figure 2.**
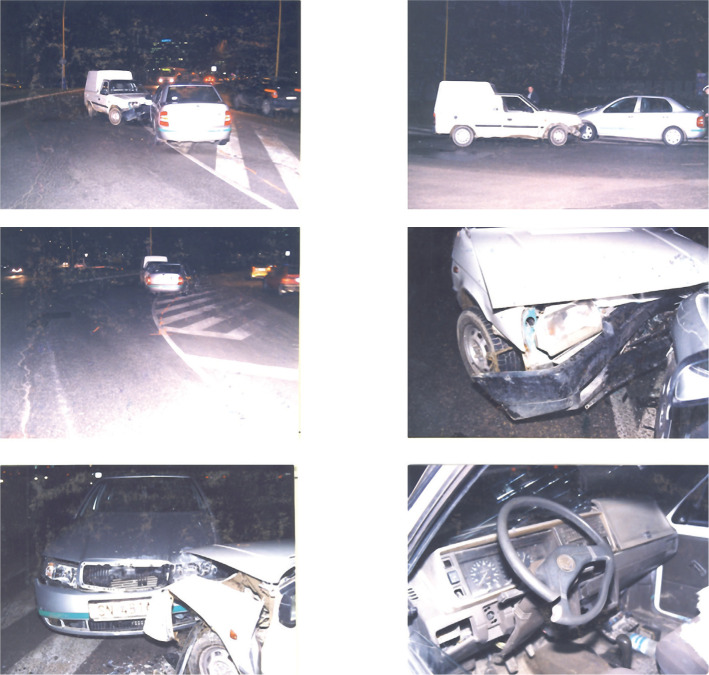
Photo documentation of the traffic accident site

The supporting documents also included the photo documentation of the accident site, which shows the situation after the accident, the extent of damage to the vehicles, and other facts identified during the inspection of the accident site (Figure 2) [3].

### Vehicle collision calculation

**Figure 3.**
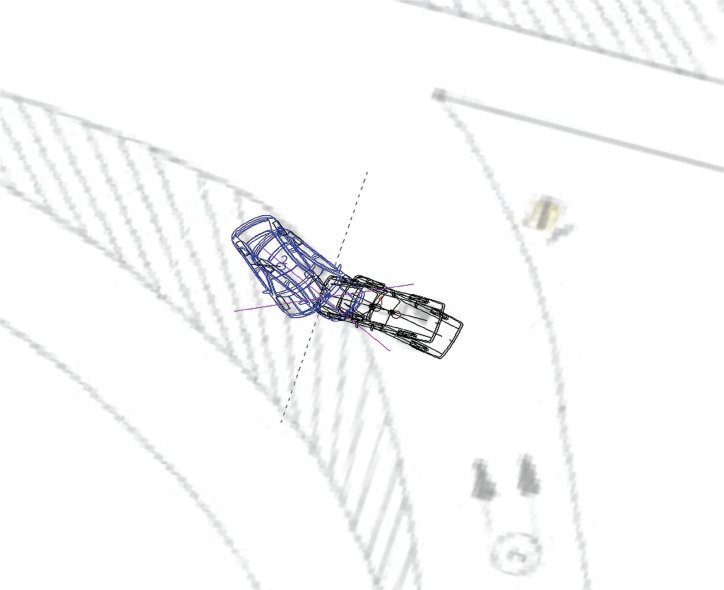
Vehicle collision calculation in the PC Crash program – initial and final positions of the vehicles.

Afterwards, a calculation of the vehicle collision was performed in the PC Crash simulation program, and a technically acceptable result was achieved: at the time of collision, the Sedan (1) was moving at a speed of 11.0 km/h, and the brake was used; the Pick-Up (2) was moving at a speed of 36.0 km/h. The calculation of the vehicle collision is demonstrated in Figure 3.

### Passenger injuries – description, parametrization, and localization

During the collision, the passenger in the Pick-up suffered the following injuries: dislocation of the left hip joint with a fracture of the left femoral head and a fracture of the posterior edge of the acetabulum, fracture of the nasal bones, and left knee contusion.

**Figure 4.**
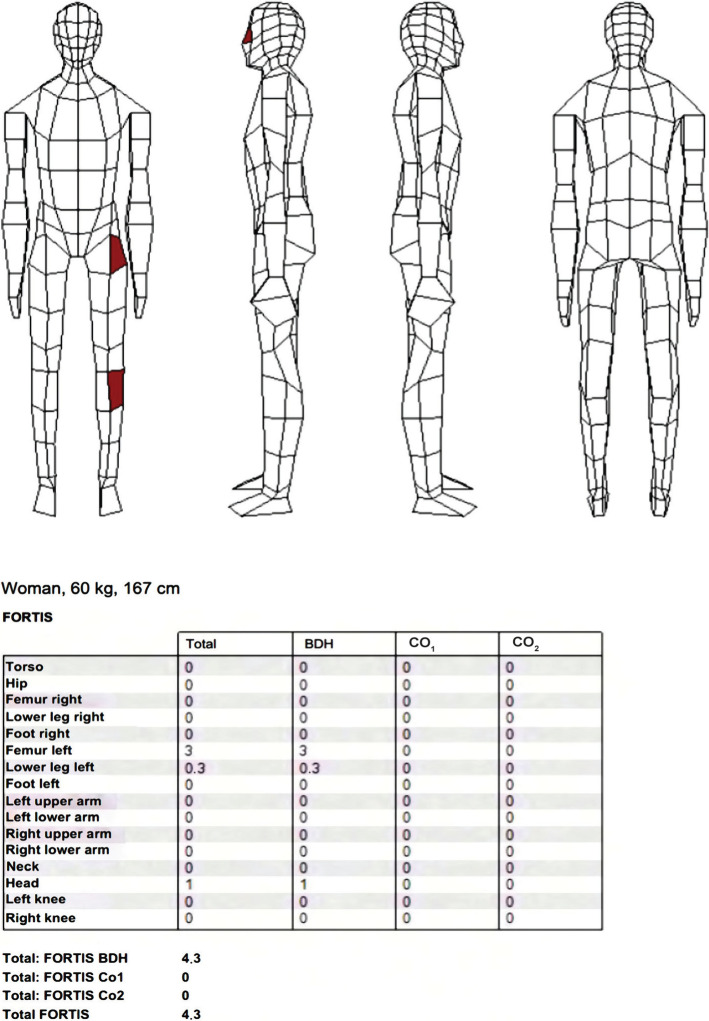
Parametrization of injuries and localization of contact places of the passenger. BDH – basic damage to health; CO_1_ – direct post-traumatic complications; CO_2_ – associated complications.

The localization of the contact places and parametrization of the injuries using the PC Fortis program was performed by a medical examiner. The FORTIS score of the detected injuries of 4.3 Fortis point (FP) is shown in Figure 4.

### Results of the calculations of a passenger dummy model and its parameters

#### Movement of an unbelted and belted passenger dummy

**Figure 5.**
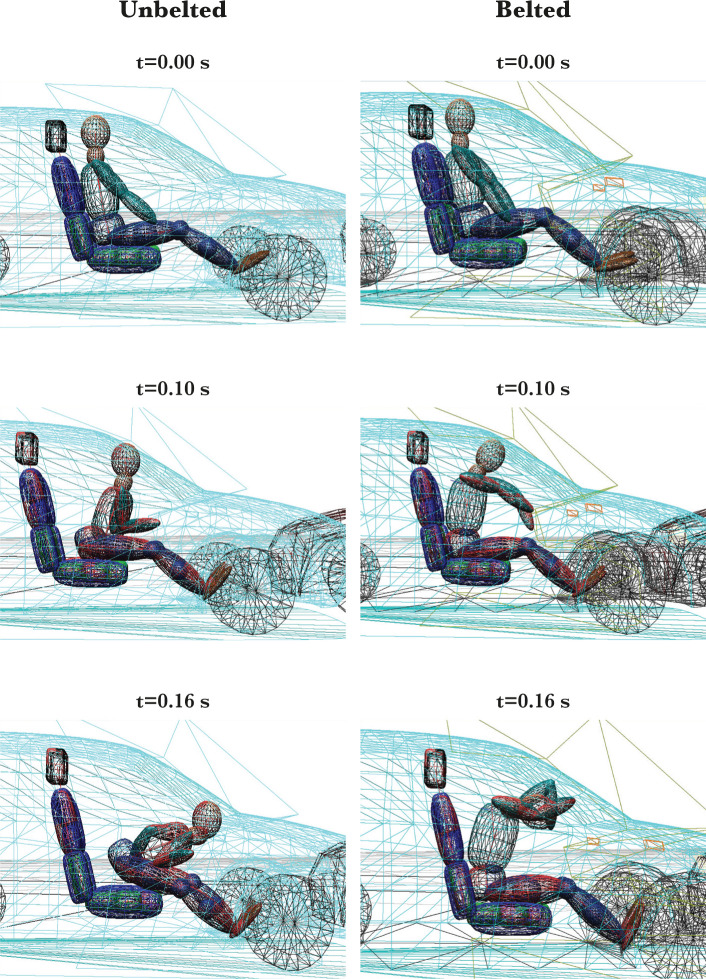
View of the passenger movement during the collision according to the calculation in the PC Crash program.

A technical expert performed this movement using a multi-body system on the PC Crash program. The figure of the calculated movement of an unbelted and belted passenger dummy is shown in Figure 5.

#### Detected parameters of the movement of a passenger dummy and its selected parts

The movement parameters of the passenger’s body and its selected parts were read from the PC Crash simulation program, which allows showing the results of the calculations also in the form of diagrams [6]. These can be exported in both graphical and numerical forms for vehicles, multi-body systems and their individual parts [1]. This option allows the “examination” of selected parameters of the movement of the whole multi-body system and its selected parts, including a mutual comparison of these parameters for the needs of medical expert examination.

**Figure 6.**
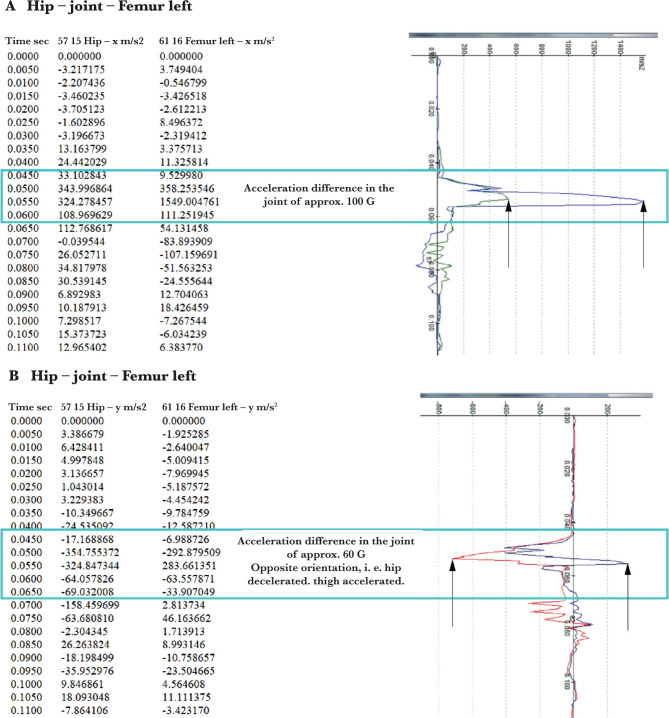

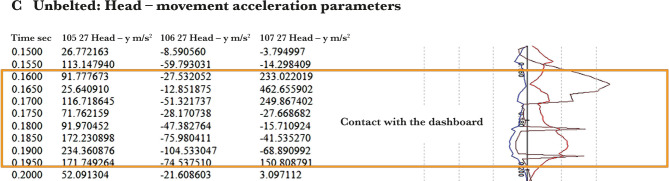
Diagrams of the movement of the left hip and thigh of the unbelted passenger with the evaluation of the difference between the applied.

Figure 6 shows a selection of diagrams of parameter values for the unbelted passenger’s body in a numerical and graphical form, and their evaluation.

The value of 100 G exceeds the general injury limit (40–80 G) [2]. The selected values were compared for an unbelted and belted passenger.

**Figure 7.**
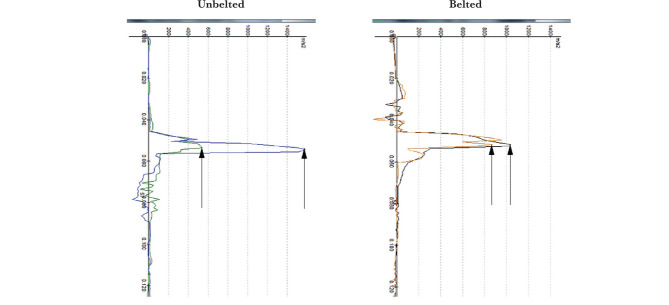
Comparison of the diagrams of the x-axis acceleration in the left hip joint.

**Figure 8.**
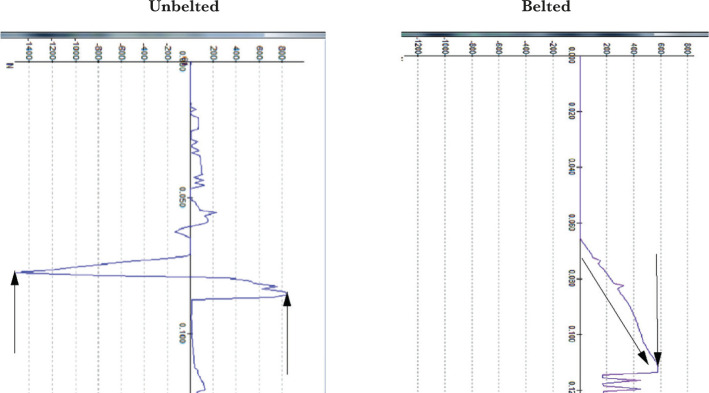
Comparison of the diagrams of contact forces affecting the left knee.

The examples of the comparison of physical parameters: acceleration in the left hip joint in the direction of the x-axis (Figure 7) and contact forces affecting the left knee during the collision with the dashboard (Figure 8) for an unbelted and belted passenger calculation model (Figures 7 and 8).

#### Medical evaluation of calculations – determination of the extent of injuries

**Figure 9.**
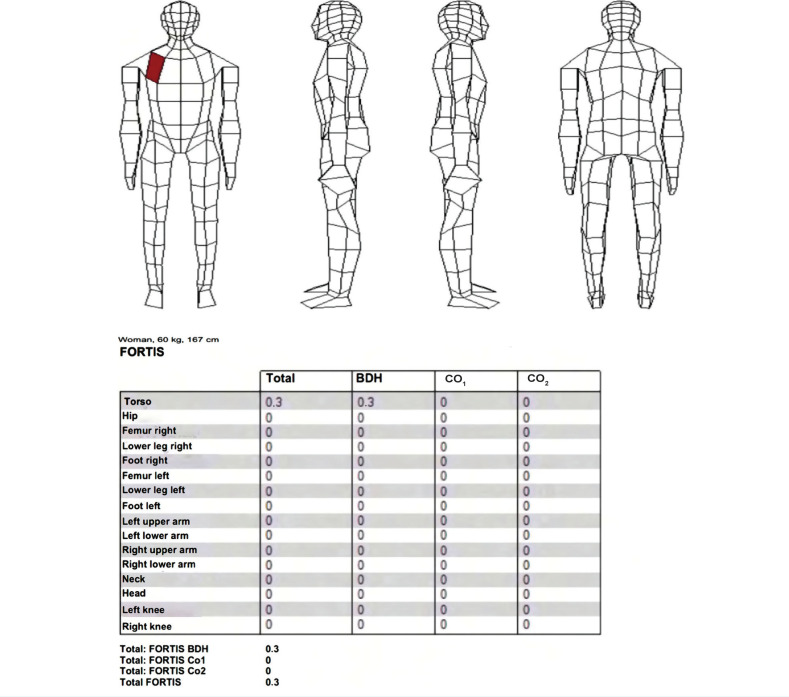
Parametrization of estimated injuries and localization of contact places of a belted passenger. BDH – basic damage to health; CO_1_ – direct post-traumatic complications; CO_2_ – associated complications.

The final values of the calculations were then presented to a medical examiner who, after considering all facts, prepared the parametrization and localization of estimated injuries to the passenger provided her seat belt had been fastened during the collision. Her FORTIS score would have been 0.3 Fortis points (Figure 9).

## Discussion

Medically defined extent and manner of injuries may help reconstruct a more detailed course of a traffic accident. These may also be very useful control values and, as described in the present article, could allow for an accurate, highly qualified estimate of potential injuries under different conditions concerning the detected circumstances of the injuries [4], through interdisciplinary procedures using selected methods of forensic medicine and forensic engineering, in particular the FORTIS system and simulation programs designed for traffic accident technical analysts. These could be, for instance, a lower impact speed, a belted passenger instead of an unbelted one etc. To do this, medical and technical theoretical premises behind the individuality of injuries and the possibility to detect these individual features, as well as the possibility of its subsequent complex assessment [5] may be used. The court accepted the conclusions of the case in question and, following these, a judgement that determined a high degree of fault in the passenger’s injuries due to failure to fasten her seat belt was rendered. The investigation and extent of injuries under different conditions that may have occurred or had been neglected is an important part of assessing the degree of fault by the court and a possible defence of the accused. The driver’s fault in a traffic accident does not always mean he/she is fully responsible for the injury, and new medical procedures in describing detected injuries bring along the mentioned possibility of proving. Obviously, in this context, there is a need to point out that, according to expert studies [8], the risks mentioned above resulting from not using seat belts mainly affect young drivers.

## Conclusion 

The authors believe that the presented methodology has considerable potential for further research not only for the investigation of traffic accidents but also for investigating other types of accidents and violent crimes against life and body by the police. It is also important to acquaint investigators and judges with the possibilities of new interdisciplinary procedures and outcomes, which should increase legal certainty for each participant in a traffic accident or a victim of crime [9]. In this paper, we documented how the extended use of simulation programs for technical analysis of traffic accidents directly affects the need to develop methods of forensic medicine, where the possibility of significant expansion of interdisciplinary procedures while using the forensic system FORTIS has arisen.

## Acknowledgments

### Conflict of interest

The authors declare that there is no conflict of interest.

### Ethical approval

No special ethics approval is needed. However, the information was shared to the ethics committee from the Faculty of Medicine, P. J. Safarik University in Kosice, Slovak Republic. This manuscript does not mention or cover any use specific or personal data concerning animals or humans or tissues.

### Authorship

NB was involved in participation and performance of medical procedures, JM, AV participated in evaluation of diagnoses, simulation in PC Crash, technical assessment, and description of cases. AO, BS conducted text processing and finalization. The authors read and approved the final manuscript.
